# Modeling propofol‐induced cardiotoxicity in the isolated‐perfused newborn mouse heart

**DOI:** 10.14814/phy2.15402

**Published:** 2022-08-03

**Authors:** Matthew B. Barajas, Aili Wang, Keren K. Griffiths, Linlin Sun, Guang Yang, Richard J. Levy

**Affiliations:** ^1^ Department of Anesthesiology Columbia University Medical Center New York New York USA

**Keywords:** cardiotoxicity, heart, isolated‐perfused heart, Langendorff preparation, model, newborn, propofol, propofol infusion syndrome

## Abstract

Infants and children are vulnerable to developing propofol infusion syndrome (PRIS) and young age is a risk factor. Cardiac involvement is often prominent and associated with death. However, the mechanisms of pediatric PRIS are poorly understood because of the paucity of investigation and lack of a gold standard animal model. Unfortunately, in vivo modeling of PRIS in a newborn mouse is not feasible and would be complicated by confounders. Thus, we focused on propofol‐induced cardiotoxicity and aimed to develop an ex‐vivo model in the isolated‐perfused newborn mouse heart. We hypothesized that the model would recapitulate the key cardiac features of PRIS seen in infants and children and would corroborate prior in vitro observations. Isolated perfused newborn mouse hearts were exposed to a toxic dose of propofol or intralipid for 30‐min. Surface electrocardiogram, ventricular contractile force, and oxygen extraction were measured over time. Real‐time multiphoton laser imaging was utilized to quantify calcein and tetramethylrhodamine ethyl ester fluorescence. Propidium iodide uptake was assessed following drug exposure. A toxic dose of propofol rapidly induced dysrhythmias, depressed ventricular contractile function, impaired the mitochondrial membrane potential, and increased open probability of the permeability transition pore in propofol‐exposed hearts without causing cell death. These features mimicked the hallmarks of pediatric PRIS and corroborated prior observations made in isolated newborn cardiomyocyte mitochondria. Thus, acute propofol‐induced cardiotoxicity in the isolated‐perfused developing mouse heart may serve as a relevant ex‐vivo model for pediatric PRIS.

## Graphical abstract

Propofol (2,6‐diisopropylphenol) infusion syndrome (PRIS) was first described in the early 1990s in several critically ill children. Using a Langendorff preparation, we found that a toxic dose of propofol rapidly induced dysrhythmias, depressed ventricular contractile force, significantly decreased the force‐frequency product, uncoupled mitochondrial respiration, impaired mitochondrial membrane potential (ΔΨ), and increased open probability of the mitochondrial permeability transition pore (mPTP) in propofol‐exposed hearts. Our model recapitulated the key cardiac features of PRIS seen in infants and children and, thus, may serve as a valid and relevant ex‐vivo model for propofol‐induced cardiotoxicity in the developing heart.

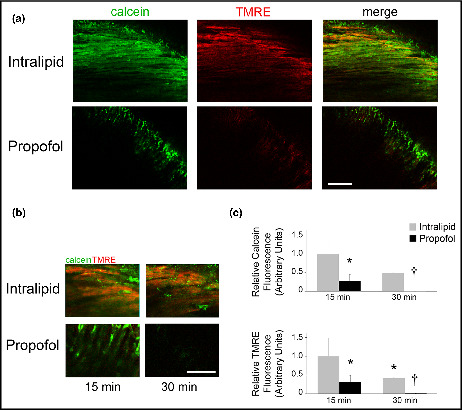

## 1 INTRODUCTION

Propofol (2,6‐diisopropylphenol) infusion syndrome (PRIS) was first described in the early 1990s in several critically ill children who were administered the drug for greater than 48 h (Bray, 2002; Parke et al., 1992). The clinical features of PRIS differ between children and adults and young age is thought to be a risk factor (Bray, 2002). However, the pathophysiological mechanisms of PRIS remain poorly understood in infants and children. In recent investigation, we exposed isolated newborn cardiomyocyte mitochondria to toxic concentrations of propofol or equal volume of intralipid (the solvent present within the clinical propofol formulation) (Barajas et al., 2022). We found that propofol uncoupled mitochondria in the developing murine heart by inducing excessive proton leak and interfered with electron transport at the level of coenzyme Q (Barajas et al., 2022). Unfortunately, a key limitation of this work is that these findings were made using an in vitro experimental approach. Thus, further study using a relevant animal model of pediatric PRIS is an obvious next step to advance our understanding.

Although propofol‐induced cytotoxicity has been the focus of many preclinical studies, the field currently lacks a gold standard animal model of PRIS. In vitro exposure is a commonly used method to characterize the toxicological effects of propofol because of its simplicity, ease, limited cost, and short timeline. However, it is not clear if the effects of such exposures translate to the clinical scenario. More complex clinically relevant models have been established, yet, entail continuous administration of high doses of propofol to mechanically ventilated and instrumented animals over several hours or days (Campos et al., 2016; Ypsilantis et al., 2007). Unfortunately, modeling PRIS in a newborn mouse in this manner is not feasible and would be complicated by introducing multiple confounding variables. Therefore, an alternative strategy is necessary.

Bradyarrhythmias, cardiac conduction abnormalities, and profound myocardial failure are central features of PRIS in children, associated with death in the majority of pediatric cases (Bray, 1998, 2002; Parke et al., 1992). Thus, in this work, we focused on propofol‐induced cardiac toxicity and aimed to develop an ex‐vivo model in the isolated‐perfused newborn mouse heart. Using a Langendorff preparation, we found that a toxic dose of propofol rapidly induced dysrhythmias and depressed ventricular contractile force. Furthermore, we detected in situ evidence for impaired mitochondrial membrane potential (ΔΨ) and open probability of the mitochondrial permeability transition pore (mPTP) in propofol‐exposed hearts. Our findings recapitulated the key cardiac features of PRIS seen in infants and children and corroborated our prior in vitro observations. Thus, our Langendorff preparation may serve as a valid and relevant ex‐vivo model for propofol‐induced cardiotoxicity in the developing heart.

## 2 MATERIALS AND METHODS

### 2.1 Animals

Care was in accordance with NIH and CUMC IACUC guidelines. The research was prospectively reviewed and approved by the CUMC IACUC. 6–8‐week old C57Bl/6N mice were acquired (Charles River) and bred to yield male pups. Postnatal day 10–13 was chosen to model a timepoint in human infancy (Clancy et al., 2007; Hornig et al., 2004; Klintsova et al., 2007).

### 2.2 Isolated‐perfused newborn mouse heart preparation

Mouse pups were anesthetized with intraperitoneal pentobarbital (70 mg/kg) and heparinized (10 kU/kg ip). The heart was rapidly excised and the aorta was cannulated as previously described (Barajas et al., 2020). Retrograde perfusion was initiated at constant flow (2.5 ml/min) with a modified Krebs–Henseleit buffer containing (mmol/L) NaCl 120, KCl 4.7, MgSO_4_ 1.2, KH_2_PO_4_ 1.2, CaCl_2_ 1.25, NaHCO_3_ 25, and glucose 11. Non‐recirculating buffer was maintained at pH 7.4 equilibrated with 95% O_2_–5% CO_2_ at 37°C. Hearts were allowed to stabilize for 30 min. Ventricular contractile force was measured in the longitudinal axis using a 5–0 silk suture connected between the apex and a force displacement transducer (ADInstruments). Diastolic tension was adjusted to 1–2 g. Electrocardiogram (ECG) was captured using surface electrodes. Heart rate, rhythm, and ventricular tension were continuously recorded with an analog‐to‐digital converter system (Power Lab 4SP, ADInstruments). Exclusion criteria were enforced as previously described (Barajas et al., 2020).

Coronary inflow oxygen tension (PaO_2_), coronary effluent oxygen tension (PvO_2_), and lactate were measured (GEM Premier 4000, Instrumentation Laboratory) in fluid aspirated into gas‐tight syringes from just proximal to the aortic cannula and from within the right atrium (using a 24‐gauge catheter), respectively. Measurements were obtained immediately following sampling to minimize atmospheric contamination. Percent oxygen extraction was calculated as 100 × ([PaO_2_ − PvO_2_]/PaO_2_). Myocardial oxygen consumption (MV̇O_2_) was calculated as (coronary flow rate per gram) × (PaO_2_ − PvO_2_) x oxygen solubility at 760 mmHg. Oxygen solubility was considered to be 24 μl/ml H_2_O at 37°C.

### 2.3 Drug exposure

Following stabilization, each heart was randomly exposed to 400 μM propofol (Diprivan, Fresenius Kabi) or equal volume 10% intralipid (Sigma‐Aldrich) for 30‐min continuously. Therapeutic concentrations of propofol range between 5–30 μM in plasma and 60–90 μM in tissue (Krajčová et al., 2015; Shortal et al., 2018). Propofol concentrations of 100–400 μM have been used previously to model for a toxic propofol exposure (Barajas et al., 2022; Vanlander et al., 2015; Weaver et al., 2001). Thus, we exposed hearts to the highest concentration of propofol (400 μM) to induce profound cardiotoxicity. Intralipid was used as a control vehicle because it is the solvent within the clinical propofol formulation (USP, 2022). Specifically, the propofol emulsion contains 10% intralipid (100 mg/ml soybean oil [a refined mixture of long‐chain fatty acids], glycerol, and egg lecithin) (USP, 2022). Thus, hearts exposed to 10% intralipid (0.07% final concentration) served as vehicle‐exposed controls. With a coronary flow rate of 2.5 ml/min, propofol‐exposed hearts were administered a total dose of 5.34 mg. Based on body weight, this cumulative dosage approximated the amount of propofol administered to a mouse over a 24‐h period during a target‐controlled infusion (60 mg/kg/h) and was equivalent to the cumulative weight‐based dose of propofol administered to rabbits in a long‐term model of PRIS (Shortal et al., 2018; Ypsilantis et al., 2007). Therefore, each propofol‐exposed mouse heart was administered a toxic dosage of propofol over 30‐min.

### 2.4 Real‐time multiphoton laser imaging

Calcein‐acetoxymethyl (AM) ester (Sigma‐Aldrich) and tetramethylrhodamine ethyl ester (TMRE) (Sigma‐Aldrich) were prepared in DMSO as 4 and 1 mM stock solutions, respectively. Cobalt chloride (CoCl_2_) (Sigma‐Aldrich) and 2,3‐butanedione monoxime (BDM) (Sigma‐Aldrich) were prepared in modified Krebs–Henseleit buffer. Following stabilization, isolated‐perfused hearts were loaded with calcein‐AM (5 μM) and TMRE (20 μM) followed by a 30‐min washout. Hearts were placed under a two‐photon microscope (Scientifica Hyperscope) and temperature was maintained at ~37°C. The laser was tuned to 940 nm. All imaging was performed using a 1.05–numerical aperture (NA) 25× objective lens to enable 100× high‐magnification (104 × 104 μm; 512 × 512 pixels; 1‐μm step) images for cardiomyocyte analysis. Hearts were then exposed to either propofol or 10% intralipid and simultaneously perfused with BDM (10 mM)(to prevent contraction‐induced movement) and CoCl_2_ (2 mM) to quench calcein fluorescence.

Ventricular cardiomyocytes were easily identified and images were obtained after 15‐ and 30‐min of exposure. Cardiomyocytes in 3–4 imaged fields per mouse were identified and arbitrary calcein and TMRE fluorescence were quantified (ImageJ) (range, 0–255, 8‐bit) within each region of interest. Calcein and TMRE fluorescence were expressed in arbitrary units and intralipid values at the 15‐min mark were arbitrarily set to equal 1.

### 2.5 Propidium iodide uptake

Isolated‐perfused hearts were exposed to either propofol or intralipid for 30‐min. Ischemia‐reperfused hearts served as positive controls and were exposed to 30‐min of warm global ischemia followed by 30‐min of reperfusion. Following drug exposure or reperfusion, hearts were loaded with 1.5 mM propidium iodide (PI)(Sigma‐Aldrich), perfused with 4% paraformaldehyde, and post‐fixed for 24 h at 4°C. Paraffin embedded hearts were cut into 6‐μm sections, slide mounted, and imaged at 10× (Nikon A1 Confocal Microscope System, Nikon Instruments, Inc.). Excitation/emission settings were 540/608 nm. The number of myocardial PI‐positive nuclei were quantified in a blinded fashion (performed by A.W.) within 3–4 sections per mouse heart (ImageJ).

### 2.6 Statistical analysis

Statistical analysis was performed using GraphPad Prism 9 (GraphPad Software). Data are presented as means ± SD. Sample number for each experiment is indicated in figure legends. A sample size of 6 per group was required to detect a 20% change in heart rate between groups with a power of 80 based on an α of 0.05. Differences between and within exposed cohorts over time were assessed using a two‐way ANOVA with repeated measure and Tukey's post hoc test. Fisher's exact test was used to assess for the association between onset of arrhythmia and drug exposure. One‐way ANOVA was used to assess for differences in PI uptake. Significance was set at *p* < 0.05.

## 3 RESULTS

### 3.1 Effects of propofol on heart rate, cardiac rhythm, and ventricular function

**FIGURE 1:**
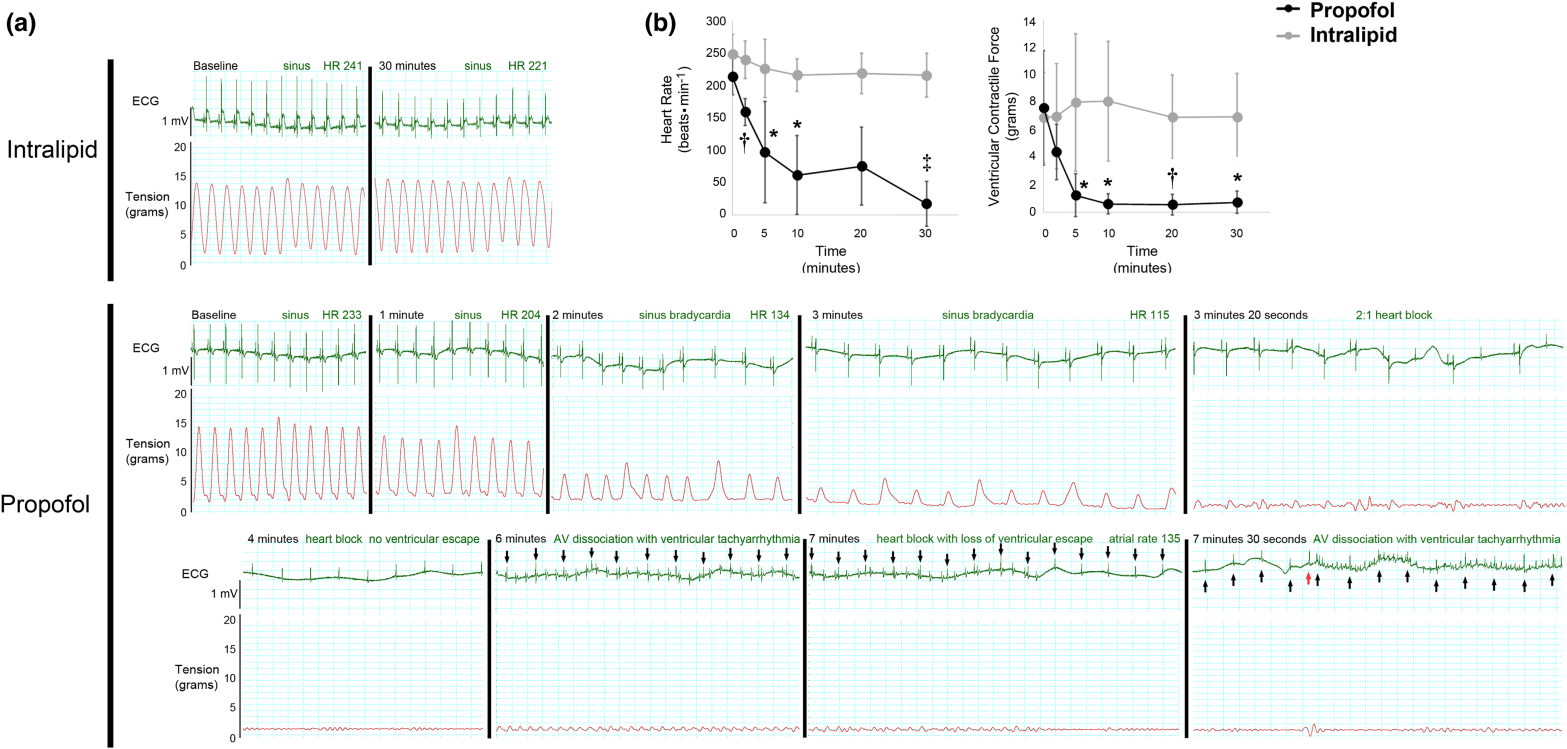
Propofol impairs heart rate, cardiac rhythm, and ventricular contractile force. (a) Representative tracings of surface electrocardiogram (ECG) and ventricular contractile force (tension) over time. Tracings captured prior to exposure (baseline) and at various time points during exposure are depicted. Heart rate (HR) is provided in beats per minute. Black arrows indicate P waves in the propofol‐exposed ECG tracing while the red arrow indicates a dissociated ventricular beat. (b) Heart rate and ventricular contractile force over time. Values are means ± SD. *n* = 6–8 per group. *p* values were calculated by two‐way ANOVA with repeated measures. *p < 0.05, ^†^ *p* < 0.01, ^‡^ *p* < 0.001 versus time‐matched intralipid values.

Following aortic cannulation and stabilization, we continuously exposed isolated‐perfused newborn hearts to a toxic dose of propofol or equal volume of intralipid for 30‐min. We measured heart rate (HR), characterized cardiac rhythm, and quantified ventricular contractile force in the longitudinal axis prior to and during exposure. There was no difference in baseline parameters between groups prior to exposure (Table S1) and the preparation remained stable throughout the study period (Figure S1). Intralipid caused minimal changes in HR, rhythm, and ventricular contractile force over time (Figure 1). Propofol rapidly induced bradycardia and significantly depressed ventricular contractile force (Figure 1). Bradycardia progressed to heart block in the majority of propofol‐exposed hearts with periods of ventricular tachyarrhythmia or lack of a ventricular escape during atrioventricular dissociation (Figure 1, Table 1). Asystole manifested in one of the propofol‐exposed hearts (Table 1). Onset of arrhythmia was significantly associated with propofol exposure. Importantly, the defects in HR, rhythm, and contractile force caused by propofol recapitulated the key cardiac features of pediatric PRIS and persisted throughout the exposure in all hearts (Bray, 1998, 2002; Parke et al., 1992). However, these effects were completely reversible and HR, rhythm, and contractile force normalized several minutes following cessation of propofol (Figure S2).

**TABLE 1:** Arrhythmias in isolated perfused newborn hearts before and after exposure to either intralipid or propofol

Exposure	Sinus bradycardia	Heart block	AV dissociation with ventricular tachyarrhythmia	Ventricular tachycardia (without AV block)	Asystole
Pre‐exposure					
Intralipid	0 (0)	0 (0)	0 (0)	0 (0)	0 (0)
Propofol	0 (0)	0 (0)	0 (0)	0 (0)	0 (0)
Post‐exposure					
Intralipid	0 (0)	0 (0)	0 (0)	0 (0)	0 (0)
Propofol	8 (100)	7 (87.5)	7 (87.5)	1 (12.5)	1 (12.5)

*Note*: Values represent number of hearts. Percentages of hearts are shown in parentheses. Onset of arrhythmia was significantly associated with propofol exposure (*p* < 0.001 as assessed with Fisher's exact test). *n* = 6 intralipid‐exposed hearts, 8 propofol‐exposed hearts. AV indicates atrioventricular. Abbreviation: AV indicates atrioventricular.

### 3.2 Myocardial oxygen consumption remains unchanged during propofol exposure

**FIGURE 2:**
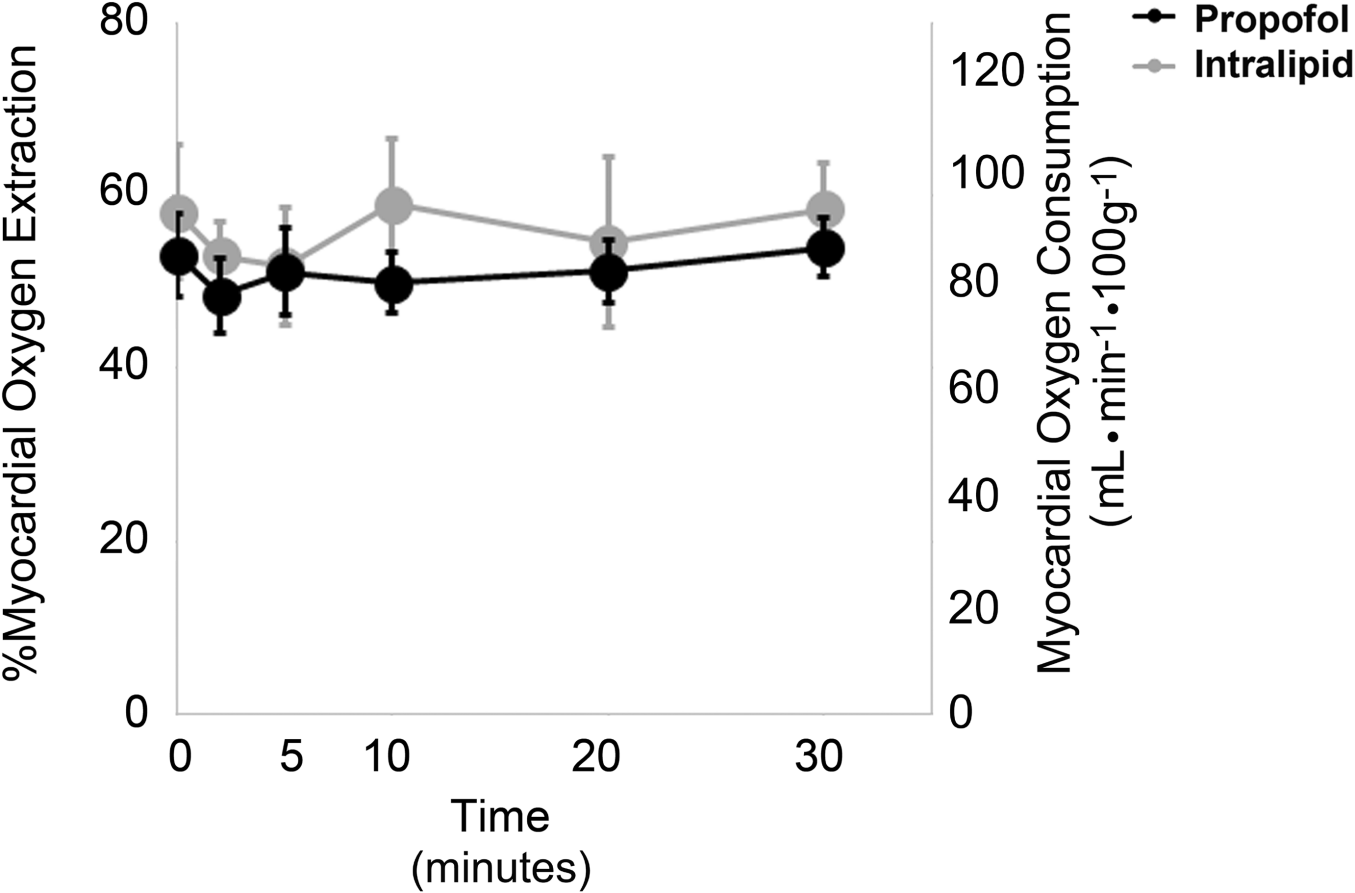
Percent myocardial oxygen extraction and oxygen consumption over time. Values are means ± SD. *N* = 6–8 per group. Significance was assessed with two‐way ANOVA with repeated measures.

In prior work, we found that propofol uncoupled mitochondrial respiration and induced excessive proton leak in vitro (Barajas et al., 2022). Thus, we next quantified myocardial oxygen extraction and calculated myocardial oxygen consumption (MV̇O_2_) to determine the in situ effect of exposure. There was no significant change over time in the percentage of oxygen extracted or in MV̇O_2_ within or between groups (Figure 2). Furthermore, lactate levels were <0.3 mmol/L in the aortic inflow and coronary effluent in both groups at all timepoints. Although the lack of change in MV̇O_2_ in the context of profound changes in HR, rhythm, and contractile function in propofol‐exposed hearts was intriguing, assessment of global myocardial oxygen extraction in our preparationfailed to provide clear insight into the in situ effects of propofol on cardiomyocyte mitochondrial respiration.

### 3.3 Propofol impairs ΔΨ and opens the mPTP in the isolated‐perfused heart

**FIGURE 3:**
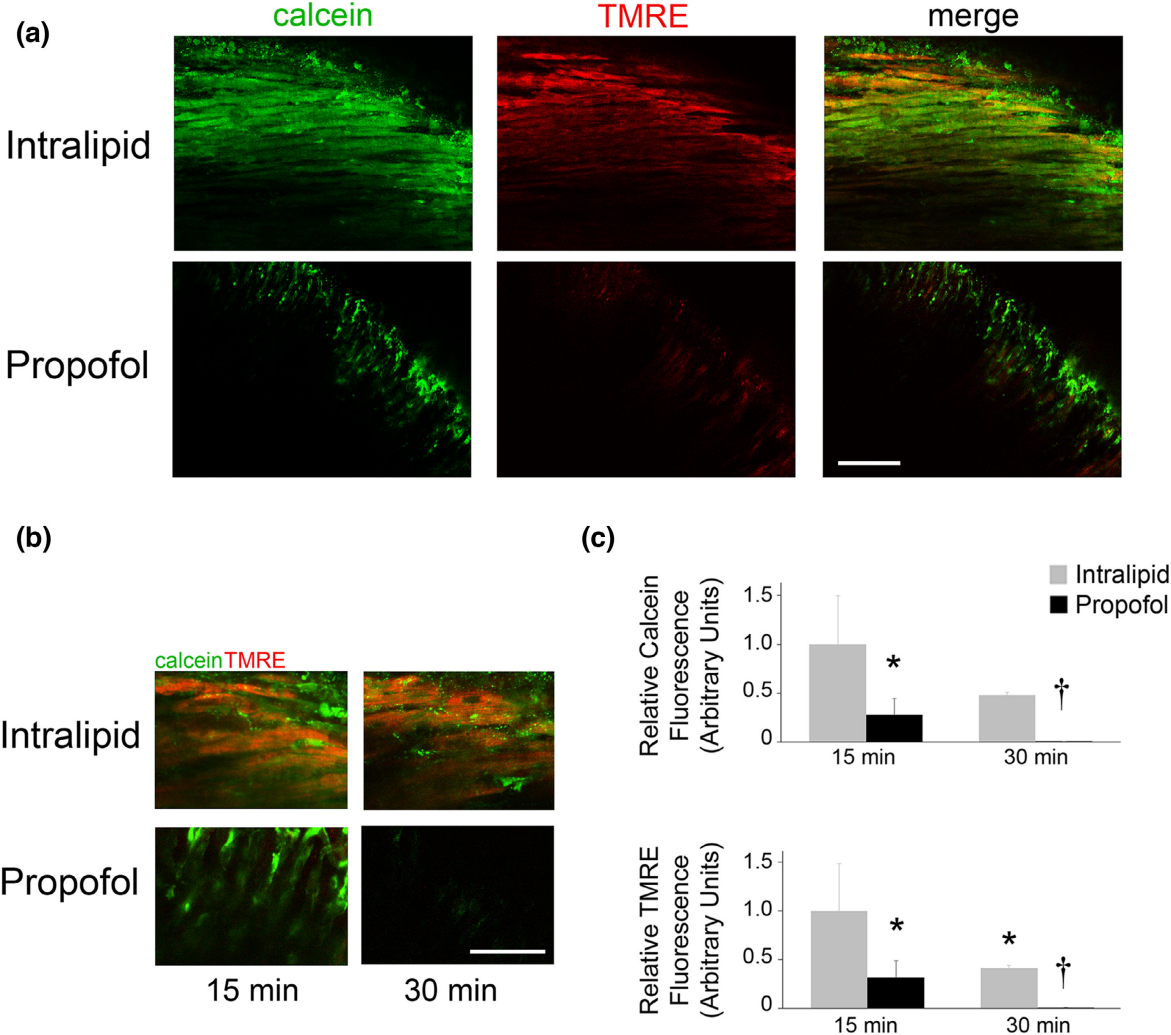
Propofol compromises ΔΨ and opens the mPTP. Real‐time multiphoton laser imaging of isolated‐perfused newborn hearts was used to measure ΔΨ and determine open probability of the mPTP during propofol or intralipid exposure. Following stabilization, hearts were loaded with calcein‐AM and tetramethylrhodamine ethyl ester (TMRE) and then exposed to either propofol or intralipid for 30 min. Calcein was quenched with cobalt chloride during each exposure. Intramitochondrial calcein (green) and actively respiring mitochondria (TMRE; red fluorescence) were identified within ventricular cardiomyocytes. Nuclei can be seen as circular voids. (a) Representative images obtained at 25× magnification after 15 min of exposure. Scale bar is 100 μm. (b) Representative merged images obtained at 100× magnification after 15 and 30 min of exposure. Scale bar is 50 μm. (c) Graphical quantification of myocardial calcein and TMRE fluorescence over time is shown. Intralipid values at the 15‐min mark were arbitrarily set to 1. *n* = 3 biological replicates per exposure group per time point. Calcein and TMRE fluorescence within 3–4 imaged fields per replicate were quantified. *p* values were calculated using two‐way ANOVA with repeated measures. *p < 0.05, ^†^ *p* < 0.01 versus intralipid values at the 15‐min mark.

The combination of proton leak and limited capacity to oxidize substrate are thought to prevent propofol‐exposed cardiomyocyte mitochondria from generating an adequate ΔΨ (Barajas et al., 2022). We previously identified multiple sources of propofol‐induced leak in exposed mitochondria, including the mPTP (Barajas et al., 2022). Thus, we next performed real‐time multiphoton laser imaging of isolated‐perfused newborn hearts to measure ΔΨ and determine open probability of the mPTP during propofol or intralipid exposure. We assessed for opening of the mPTP in situ by quantifying intramitochondrial calcein fluorescence following cobalt‐mediated quenching (Petronilli et al., 1999). Actively respiring mitochondria were simultaneously labeled with tetramethylrhodamine ethyl ester (TMRE) for co‐localization and for real‐time dynamic quantification of ΔΨ (Matsumoto‐Ida et al., 2006). Fluorescence waned somewhat over time in intralipid‐exposed hearts, possibly as a result of the obligatory ischemia/reperfusion inherent to the process of establishing the Langendorff preparation. However, robust intramitochondrial calcein and TMRE fluorescence was seen throughout the duration of exposure, indicating closed probability of the mPTP and relative maintenance of ΔΨ (Figure 3). In contrast, propofol‐exposed hearts demonstrated significant loss of cardiomyocyte calcein and TMRE fluorescence over time (Figure 3). The findings indicated a marked ΔΨ decline in propofol‐exposed cardiomyocyte mitochondria and opening of the mPTP. Importantly, these results were consistent with our prior in vitro observations and provided further evidence that propofol interferes with the ability of cardiac mitochondria to generate and maintain ΔΨ and opens leak channels, such as the mPTP.

### 3.4 Lack of cell death in propofol‐ and intralipid‐exposed hearts

**FIGURE 4:**
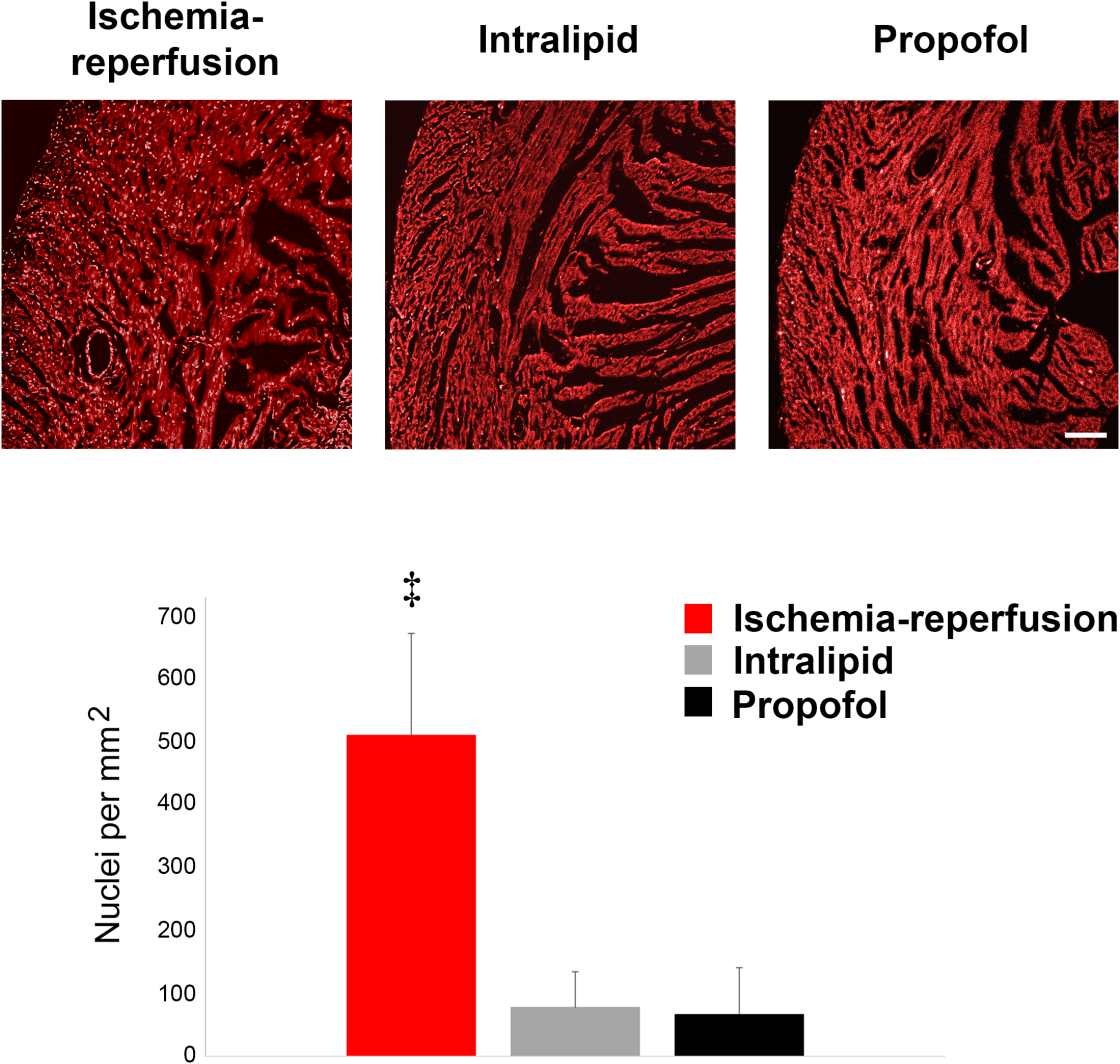
Lack of cell death in propofol‐ and lipid‐exposed hearts. Propidium iodide (PI) uptake by cardiomyocyte nuclei was assessed and quantified following exposure to propofol or intralipid. Ischemia‐reperfused hearts served as positive controls. Representative images obtained at 10× magnification are shown (above). Scale bar is 100 μm. Bright punctate PI‐positive nuclei are easily seen. Graphical quantification of the number of myocardial PI‐positive nuclei is shown (below). *n* = 3 biological replicates per exposure group. PI fluorescence within 3–4 imaged fields per replicate was quantified. *p* values were calculated using one‐way ANOVA. ^‡^ *p* < 0.001 versus propofol‐ and intralipid‐exposed hearts.

Bradyarrhythmias and progressive myocardial failure are overt and key clinical features of pediatric PRIS (Bray, 1998, 2002; Parke et al., 1992). However, histological cardiac examination has yielded relatively unremarkable findings in the majority of autopsies (Parke et al., 1992). Likewise, only myocardial infiltration with acute inflammatory cells and limited foci of myofibril degeneration or early rhabdomyolysis were seen in a lethal rabbit model of PRIS (Ypsilantis et al., 2007). Therefore, we assessed for cell death (or lack thereof) in isolated‐perfused hearts during propofol or intralipid exposure by quantifying propidium iodide (PI) uptake within cardiomyocyte nuclei as an early indicator of plasma membrane disruption (Matsumoto‐Ida et al., 2006). Hearts were perfused with PI at the end of the 30‐min exposure and the number of PI‐positive nuclei were quantified. Ischemia‐reperfused hearts exposed to 30‐min of warm ischemia followed by 30‐min of reperfusion served as positive controls. As expected, ischemia‐reperfused hearts demonstrated a significant number of PI‐positive nuclei, indicating early cell death (Figure 4). However, both propofol‐ and intralipid‐exposed hearts demonstrated markedly less PI uptake (Figure 4). Furthermore, there was no significant difference in the number of PI‐positive nuclei between propofol‐ and lipid‐exposed groups (Figure 4). The findings suggested a lack of myocardial cell death following propofol or intralipid exposure.

## 4 DISCUSSION

PRIS is a potentially lethal consequence of propofol administration that manifests with lactic acidosis, cardiac arrhythmias, myocardial failure, rhabdomyolysis, hyperkalemia, hepatomegaly, hyperlipidemia, renal failure, and fever (Hemphill et al., 2019; Vanlander et al., 2015). Young age is thought to be a risk factor and the condition was first identified in critically ill children sedated via a continuous propofol infusion in the ICU (Bray, 2002; Parke et al., 1992). However, the mechanisms of pediatric PRIS are poorly understood largely because of the paucity of studies in infants and children and the lack of preclinical investigation in developing animals. Thus, we attempted to develop an ex‐vivo model of propofol‐induced cardiotoxicity in the isolated‐perfused newborn mouse heart. We focused on the heart given that most pediatric cases of PRIS result in bradyarrhythmias, conduction abnormalities, and cardiac failure and that such cardiac manifestations are commonly associated with mortality (Bray, 1998, 2002; Parke et al., 1992). We found that a toxic dose of propofol rapidly induced dysrhythmias, depressed ventricular contractile function, impaired ΔΨ, and increased open probability of the mPTP in propofol‐exposed hearts. Although our model failed to provide in situ evidence of excessive proton leak, it mimicked the hallmark features of pediatric PRIS and corroborated other observations made in isolated newborn cardiomyocyte mitochondria (Barajas et al., 2022; Bray, 1998, 2002; Parke et al., 1992). Thus, our Langendorff preparation may serve as a relevant ex‐vivo model of propofol‐induced cardiotoxicity in the isolated‐perfused developing mouse heart.

Prior preclinical investigations have utilized in vitro and in vivo approaches to model for PRIS. The most clinically relevant in vivo models attempt to mimic the clinical scenario by continuously administering high doses of propofol to mechanically ventilated and instrumented animals over several hours or days (Campos et al., 2016; Ypsilantis et al., 2007). These complex models require constant assessment of anesthetic depth, continuous monitoring of vital signs, maintenance of euthermia, and frequent analysis of arterial blood gas values to enable correction of acid–base status, serum electrolytes, and blood glucose (Campos et al., 2016; Ypsilantis et al., 2007). Rabbits are considered to be ideal for such models given the feasibility of the approach and the fact that they develop asystole and other key features of PRIS after a prolonged propofol infusion (Campos et al., 2016). However, currently there are no clinically relevant animal models of pediatric PRIS.

In contrast to the rabbit, the mouse has gained popularity as the species of choice when modeling human disease given its low maintenance costs, shorter breeding times, high progeny numbers, small size, ease of manipulation, availability of inbred strains, and ability to be genetically manipulated (Liao et al., 2012). Unfortunately, in vivo modeling of PRIS in an infant mouse is not feasible and would be complicated by confounding variables. Thus, we opted to develop an ex‐vivo model of propofol‐induced cardiotoxicity in the isolated‐perfused newborn mouse heart given that the Langendorff preparation is a powerful investigative tool to study pharmacological responses in the absence of confounders (Liao et al., 2012). In our model, we exposed isolated‐perfused hearts to murine dosages of propofol that approximated cumulative weight‐based toxic doses known to cause PRIS (Shortal et al., 2018; Ypsilantis et al., 2007). However, we recognize that the acute timeline of exposure did not mimic the clinical scenario and hearts were exposed to a very high dosage of propofol over a short period of time. Despite these limitations, our ex‐vivo approach resulted in overt phenotypic consequences that were strikingly similar to the human condition. Although questions remain about how our findings translate to the human scenario, our model likely has clinical relevance and will serve as a useful foundation for future investigation given that acute preclinical exposures are well‐accepted strategies for elucidating cellular mechanisms of chronic toxicity and that PRIS was recently described in a relatively healthy infant following a single bolus of propofol (Mancilla et al., 2019; Michel‐Macías et al., 2018).

The exact pathophysiology of PRIS remains poorly defined, however, a growing body of evidence suggests a mitochondrial etiology (Barajas et al., 2022; Hemphill et al., 2019; Krajčová et al., 2015; Vanlander et al., 2015). Using an in vitro approach, we previously found that high concentrations of propofol induced mitochondrial proton leak in the developing murine heart by opening leak channels (such as the mPTP), interfered with electron transport at the level of coenzyme Q, and prevented mitochondria from generating and maintaining an adequate ΔΨ (Barajas et al., 2022). In the current study, we found that a toxic dose of propofol markedly decreased ΔΨ over time in the isolated‐perfused heart and increased open probability of the mPTP. Thus, our ex‐vivo model corroborated our previous key in vitro findings.

Surprisingly, postmortem analyses of hearts of infants and children who died from PRIS have demonstrated relatively unremarkable histological findings (Parke et al., 1992). Similarly, only small foci of cardiac myofibril degeneration were seen in a lethal rabbit model of PRIS (Ypsilantis et al., 2007). Consistent with this, we found minimal PI uptake within cardiomyocyte nuclei in propofol‐exposed hearts. Although PI uptake can be delayed during cell death and we have not ruled out early apoptosis, ventricular dysfunction in our model did not coincide with late‐stage or widespread cardiomyocyte degeneration. Thus, overt myocardial cell death is not a major characteristic of propofol‐induced cardiotoxicity in the developing heart despite opening of the mPTP and collapse of ΔΨ within cardiac mitochondria. Lack of cellular degeneration in the context of profound myocardial depression suggests downregulated cardiomyocyte activity to preserve viability when ΔΨ is compromised. This type of response is classically seen with “myocardial stunning”, when transient and reversible mechanical dysfunction manifests in the absence of irreversible histological injury (Guaricci et al., 2018). In our model, HR, cardiac rhythm, and contractile function normalized several minutes following cessation of propofol exposure, suggesting a reversible process. Such reversibility has been observed clinically, with complete recovery of cardiac function in children who survived PRIS (Culp et al., 2004). Furthermore, a recent report described myocardial stunning in a 14 year old girl who experienced the acute onset of dysrhythmias and global left ventricular hypokinesis immediately following the induction of anesthesia with propofol for brain magnetic resonance imaging (Faleiro Oliveira et al., 2016). Thus, the cardiac features of pediatric PRIS could manifest as a consequence of propofol‐induced myocardial stunning in the developing heart. Further investigation will certainly be required to explore such a concept and mechanism.

In this work, we successfully developed an ex‐vivo model of propofol‐induced cardiotoxicity in the isolated‐perfused newborn mouse heart. Our model exhibited all of the central cardiac features described in infants and children with PRIS. In addition, the model provided in situ evidence of propofol‐mediated mitochondrial toxicity, corroborating our prior in vitro results. Thus, the propofol‐exposed isolated‐perfused newborn mouse heart is a valid and relevant ex‐vivo model of propofol‐induced cardiotoxicity in the developing heart. Importantly, the model will now enable us to investigate the mechanisms of PRIS‐associated cardiac failure at the organ system level. Ultimately, it may aid in the development of novel therapies to treat or prevent pediatric PRIS.

## AUTHOR CONTRIBUTIONS

Substantial contributions to conception and design, acquisition of data, or analysis and interpretation of data: MBB, AW, KKG, LS, GY, RJL. Drafting the article or revising it critically for important intellectual content: MBB, LS, GY, RJL. Final approval of the version to be published: MBB, AW, KKG, LS, GY, RJL.

## FUNDING INFORMATION

This work was supported by NINDS at NIH [R01NS112706 (RJL)], NIAAA at NIH [R01AA027108 (GY)], NIGMS at NIH [R35GM131765 (GY)], and NIA at NIH [R01AG041274 (GY)].

## CONFLICTS OF INTEREST

The authors declare no competing interests.

## Supporting information


Figure S1

Table S1

Figure S2
