# Supplementary material for: Modeling propofol‐induced cardiotoxicity in the isolated‐perfused newborn mouse heart
Source: Physiol Rep. 2022 Aug 3;10(15):e15402. doi: 10.14814/phy2.15402 (PMC9350423; doi:10.14814/phy2.15402)
Supplement: Supplementary file 1 — Figure S1 [file PHY2-10-e15402-s002.pdf]

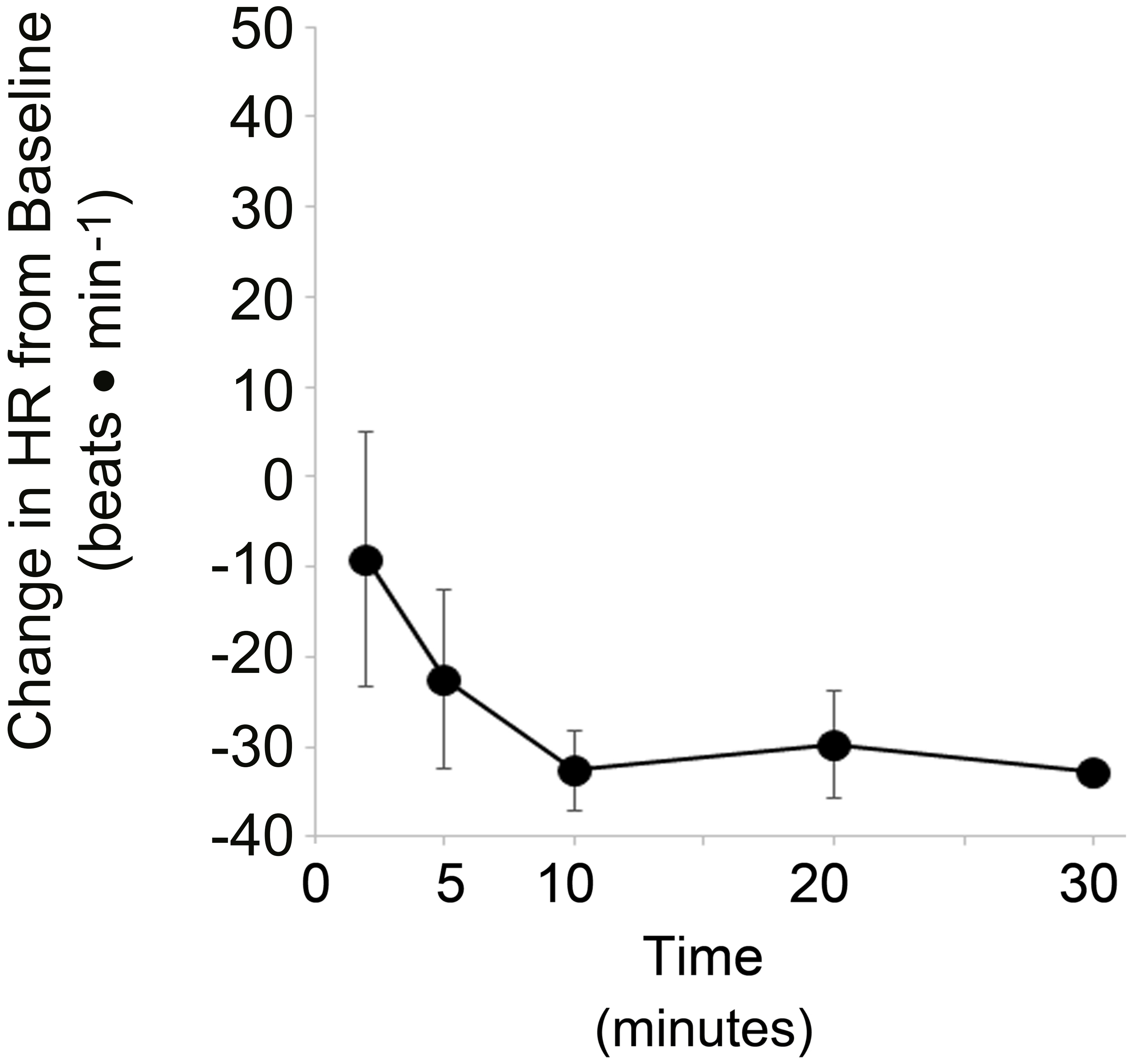

Change in HR from Baseline
(beats • min-1)
50
40
30
20
10
0
-10
-20
-30
-40
0
5
10
20
30
Time
(minutes)

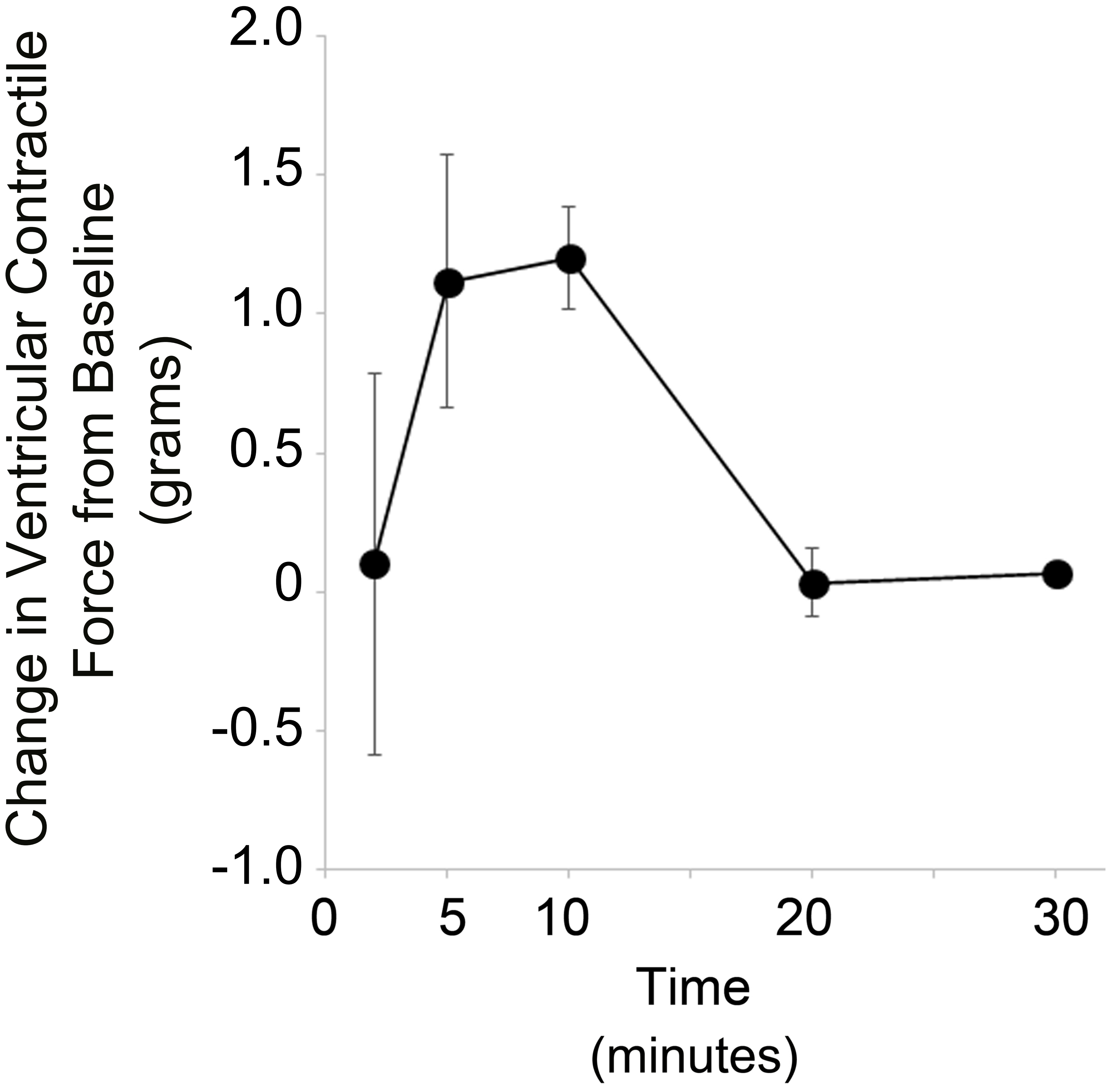

Change in Ventricular Contractile
Force from Baseline
(grams)
2.0
1.5
1.0
0.5
0
-0.5
-1.0
0
5
10
20
30
Time
(minutes)
