# Supplementary material for: Modeling propofol‐induced cardiotoxicity in the isolated‐perfused newborn mouse heart
Source: Physiol Rep. 2022 Aug 3;10(15):e15402. doi: 10.14814/phy2.15402 (PMC9350423; doi:10.14814/phy2.15402)
Supplement: Supplementary file 2 — Table S1 [file PHY2-10-e15402-s001.docx]

| **Exposure** | **Baseline HR**  **(bpm)** | **Baseline Ventricular Conractile Force (g)** | **Baseline Aortic Perfusion Pressure (mmHg)** |
| --- | --- | --- | --- |
|  |  |  |  |
| Intralipid | 249.2 ± 12.4 | 6.9 ± 1.9 | 49.3 ± 12.2 |
|  |  |  |  |
|  |  |  |  |
| Propofol | 214.4 ± 10.1 | 7.6 ± 2.1 | 47.1 ± 9.9 |
|  |  |  |  |

**Supplemental Table. Baseline parameters prior to intralipid or propofol exposure.** Values are means ± SEM. AV, atrioventricular. There was no significant difference between groups as assessed with Student’s t-test. N = 6 intralipid-exposed hearts, 8 propofol-exposed hearts. HR, heart rate; bpm, beats per minute; g, grams; mmHg, millimeters of mercury.
