# Supplementary figures and images for: Modeling propofol‐induced cardiotoxicity in the isolated‐perfused newborn mouse heart
Source: Physiol Rep. 2022 Aug 3;10(15):e15402. doi: 10.14814/phy2.15402 (PMC9350423; doi:10.14814/phy2.15402)

## Propofol exposure

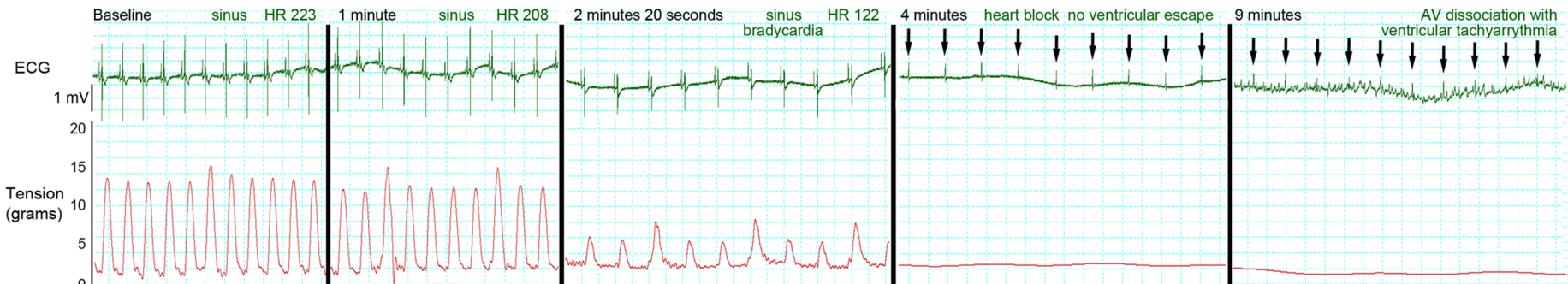

## Post-exposure

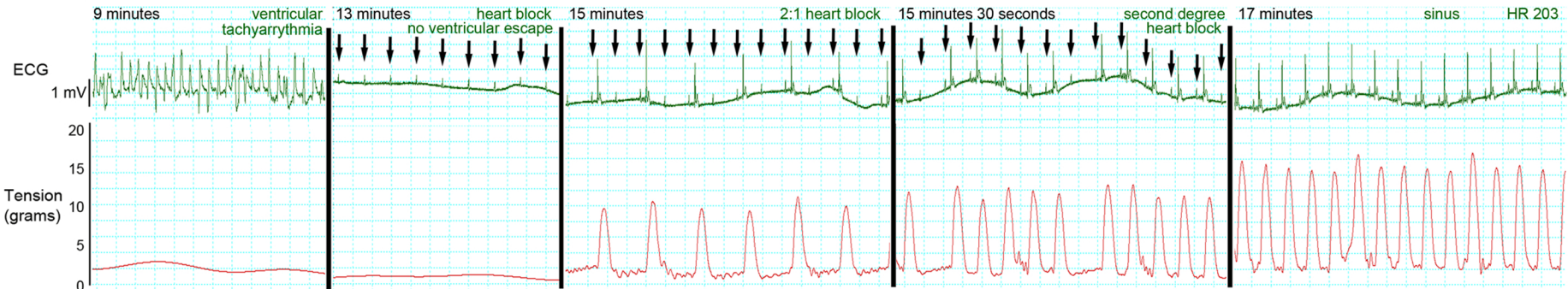

Supplement: Supplementary file 3 — Figure S2 [file PHY2-10-e15402-s003.pdf]
